# Ferulic Acid Regulates the Nrf2/Heme Oxygenase-1 System and Counteracts Trimethyltin-Induced Neuronal Damage in the Human Neuroblastoma Cell Line SH-SY5Y

**DOI:** 10.3389/fphar.2015.00305

**Published:** 2016-01-08

**Authors:** Stefania Catino, Fabiola Paciello, Fiorella Miceli, Rolando Rolesi, Diana Troiani, Vittorio Calabrese, Rosaria Santangelo, Cesare Mancuso

**Affiliations:** ^1^Institute of Pharmacology, Catholic University School of MedicineRoma, Italy; ^2^Department of Head and Neck Surgery, Catholic University School of MedicineRoma, Italy; ^3^Institute of Human Physiology, Catholic University School of MedicineRoma, Italy; ^4^Department of Biomedical and Biotechnological Sciences, School of Medicine, University of CataniaCatania, Italy; ^5^Institute of Microbiology, Catholic University School of MedicineRome, Italy

**Keywords:** biliverdin reductase, cell stress response, ferulic acid, heme oxygenase, Nrf2, SH-SY5Y, trimethyltin

## Abstract

Over the past years, several lines of evidence have pointed out the efficacy of ferulic acid (FA) in counteracting oxidative stress elicited by β-amyloid or free radical initiators, based on the ability of this natural antioxidant to up-regulate the heme oxygenase-1 (HO-1) and biliverdin reductase (BVR) system. However, scarce results can be found in literature regarding the cytoprotective effects of FA in case of damage caused by neurotoxicants. The aim of this work is to investigate the mechanisms through which FA exerts neuroprotection in SH-SY5Y neuroblastoma cells exposed to the neurotoxin trimethyltin (TMT). FA (1–10 μM for 6 h) dose-dependently increased both basal and TMT (10 μM for 24 h)-induced HO-1 expression in SH-SY5Y cells by fostering the nuclear translocation of the transcriptional activator Nrf2. In particular, the co-treatment of FA (10 μM) with TMT was also responsible for the nuclear translocation of HO-1 in an attempt to further increase cell stress response in SH-SY5Y cells. In addition to HO-1, FA (1–10 μM for 6 h) dose-dependently increased the basal expression of BVR. The antioxidant and neuroprotective features of FA, through the increase of HO activity, were supported by the evidence that FA inhibited TMT (10 μM)-induced lipid peroxidation (evaluated by detecting 4-hydroxy-nonenal) and DNA fragmentation in SH-SY5Y cells and that this antioxidant effect was reversed by the HO inhibitor Zinc-protoporphyrin-IX (5 μM). Among the by-products of the HO/BVR system, carbon monoxide (CORM-2, 50 nM) and bilirubin (BR, 50 nM) significantly inhibited TMT-induced superoxide anion formation in SH-SY5Y cells. All together, these results corroborate the neuroprotective effect of FA through the up-regulation of the HO-1/BVR system, via carbon monoxide and BR formation, and provide the first evidence on the role of HO-1/Nrf2 axis in FA-related enhancement of cell stress response in human neurons.

## Introduction

For over 40 years, since Tenhunen et al. (1969) demonstrated the heme-metabolizing activity of heme oxygenase (HO) with the formation of biliverdin (BV) and carbon monoxide (CO), and Mahin Maines – along with her group – documented the regulation and distribution of major isoforms of the protein, termed HO-1, and HO-2 (Cruse and Maines, 1988; Ewing and Maines, 1991, 1992; Maines, 1997), numerous studies have appeared in the literature showing the role of this enzyme in the modulation of biological functions, including the complex cellular response to stress in many cells and tissues (Mancuso et al., 2007; Motterlini and Foresti, 2014; Wegiel et al., 2014). Over the years, biliverdin reductase (BVR), the other enzyme that is functionally linked with HO, has been extensively studied leading to important discoveries, such as the ability to reduce BV into bilirubin (BR) using different cofactors depending on the intracellular pH (Kutty and Maines, 1981). Although the two isoforms of HO catalyze the same reaction, HO-1 is mainly involved in the adaptive response to cellular oxidative and nitrosative stress caused by an increased production of reactive oxygen and nitrogen species (ROS and RNS, respectively; Bach, 2005; Gozzelino et al., 2010), whereas HO-2 is primarily involved in the physiological regulation of heme metabolism (Maines, 1997; Maines and Panahian, 2001). Regarding BVR, in addition to the above mentioned reductase activity, recent studies have also shown the kinase activity on serine, threonine, and tyrosine residues which makes this protein unique in regulating the intracellular redox state and metabolism (Lerner-Marmarosh et al., 2005, 2007). A biological role for the HO/BVR system has been demonstrated in the central nervous system (CNS) where CO was involved in the processes of long-term potentiation (Verma et al., 1993; Zhuo et al., 1993) and neuropeptide release (Mancuso et al., 1997, 1999; Errico et al., 2010), whereas a strong antioxidant activity, through the interaction with ROS/RNS (Stocker et al., 1987; Barone et al., 2009b; Mancuso et al., 2012), and neurotrophic features (Mancuso et al., 2008) were documented for BR. On these grounds, numerous researchers have put forth the hypothesis regarding the usefulness of HO-1 and BVR up-regulation, particularly in the case of diseases caused by an excessive production of free radicals, such as neurodegenerative disorders. A common approach is the use of natural products, such as (poly)phenols, particularly abundant in fruits and vegetables, which have proved capable of increasing the expression of HO-1 in preclinical *in vitro* and *in vivo* systems (Scapagnini et al., 2011).

Among the phenolic acid derivatives, one of the most studied for its neuroprotective activity was ferulic acid (FA), particularly abundant in vegetables (aubergines, tomatoes, artichokes), fruits, grains, and some beverages (Mancuso and Santangelo, 2014). FA counteracted the oxidative/nitrosative stress due to the amyloid-β-peptide (Aβ) or other radical initiators, such as AAPH and iron/hydrogen peroxide, in neural cells with multiple mechanisms, including the activation of cell stress response (Kanski et al., 2002; Joshi et al., 2006; Perluigi et al., 2006; Picone et al., 2009). FA was also shown to potentiate the cell stress response in an *in vivo* model of neurodegeneration, namely the middle cerebral artery occlusion in rats, through the activation of both peroxiredoxin-2 and thioredoxin (Sung et al., 2014). In addition to these antioxidant effects, FA was demonstrated to contribute to the maintenance of homeostasis by modulating cellular autophagy, which is a well-known mechanism involved in the processing and elimination of misfolded proteins (Bian et al., 2013). These findings have led many researchers to hypothesize an adjuvant role for FA in neurodegenerative diseases characterized by cognitive impairment, such as Alzheimer’s disease. However, scarce evidence is present in literature concerning the neuroprotective action of FA in the event of damage due to neurotoxins, such as the trimethyltin (TMT), that causes brain damage with multiple mechanisms including the production of ROS and RNS (Gunasekar et al., 2001; Qu et al., 2011). These considerations prompted us to study the possible neuroprotective role of FA in human neuroblastoma cells, SH-SY5Y, exposed to TMT; furthermore, the research has assessed whether FA is able to enhance the HO-1/BVR system, thus reducing the neurotoxic effects of TMT in the SH-SY5Y cells.

## Materials and Methods

### Chemicals

All chemicals were purchased from Sigma–Aldrich (Milan, Italy) unless otherwise specified. FA, BR, and Zinc-protoporphyrin-IX (ZnPP or ZnPP-IX, Frontier Scientific, Logan, UT, USA) were dissolved in alkaline aqueous solution, whereas TMT in deionized water. Tricarbonyldichlororuthenium (II) (CORM-2) was dissolved in DMSO at the stock solution of 10 mM.

### Cell Culture

SH-SY5Y neuroblastoma cells were provided through the courtesy of Prof. Randall N. Pittman (Department of Pharmacology, University of Pennsylvania, Philadelphia, USA) and cultured in Minimum Eagle’s Medium (MEM, Euroclone, Pero, Italy):F12 (Gibco, Life Tecnologies, Monza, Italy) supplemented with 1X non-essential aminoacids (Euroclone), 1 mM sodium pyruvate (Gibco), 1.5 g/L sodium bicarbonate, 1% penicillin/streptomycin (Euroclone) and 10% fetal calf serum (FCS, Euroclone).

The day before the experiment, 1.2 × 10^6^ SH-SY5Y cells (10^th^–14^th^ passage) were seeded in six-multiwell plates at a density of 120,000 cells/cm^2^. After overnight incubation, cells were treated with FA (1–10 μM) for 6 h in 1% FCS medium. At the end of incubation, the medium was removed and replaced with 1% FCS medium containing TMT (10 μM) for further 24 h. In selected dose-response experiments, SH-SY5Y cells were treated either with FA (1–10 μM) for 6 h and then 24 h with plain medium or with TMT (1–10 μM) for 24 h.

### Western Blot

SH-SY5Y cells, treated as described above, were lysed in RIPA buffer (Sigma–Aldrich, Milan, Italy) for 15 min in ice. After centrifugation (12,000 rpm, 15 min, 4°C), supernatant aliquots were used to determine protein concentration using the DC protein assay kit (BIO-RAD, Segrate, Milan, Italy). Another supernatant aliquot was boiled for 3 min after the addition of 1/6 reducing loading buffer 6X. A 30 μg dose of protein was separated by SDS-polyacrylamide gel electrophoresis (SDS-PAGE) on 12% gel. Precision Plus Protein^TM^ Standards electrophoresis markers (BIO-RAD) were used as molecular mass standards. Proteins were then transferred onto nitrocellulose membranes by using the Trans-Blot^®^Turbo^TM^ Blotting System (BIO-RAD), according to the manufacturer’s instructions, and stained with Ponceau S (ICN Biochemicals, OH). Non-specific binding sites were blocked with 5% dry milk in Tris-buffered saline (TBS) and membranes were incubated overnight at 4°C with the primary rabbit anti-HO-1 (1:1000; Stressgen, Enzo Life Sciences, DBA Italia, Segrate, Milan, Italy) and anti-BVR (1:1000; Stressgen) antibodies. Membranes were then incubated with a horseradish peroxidase conjugated anti-rabbit (1:3000, BIO-RAD) IgG secondary antibody and then developed using the enhanced chemiluminescence reagents Clarity^TM^ Western ECL substrate (BIO-RAD). The bands were visualized by autoradiography using UltraCruz^TM^ autoradiography films (DBA Italia). An anti-β-actin rabbit monoclonal antibody (1:1000; Stressgen) was used to detect β-actin to confirm equal protein loading; in selected experiments, nitrocellulose membranes were stripped and then re-probed with the anti-β-actin antibody. The obtained films were evaluated for densitometry by means of an optical computed system (GEL-DOC^TM^ EM Imager, BIO-RAD). The HO-1 or BVR/β-actin ratio was calculated and expressed as a percentage compared to the control group.

### Immunofluorescence Analysis

In order to perform immunofluorescence for 4-HNE, Nrf2, and HO-1, 300,000 cells, seeded in glass coverslips (10 mm diameter) were treated as previously described. In selected experiments to evaluate 4-HNE and DNA fragmentation, cells were incubated with ZnPP (5 μM) in the presence of FA (10 μM for 6 h) and TMT (10 μM for further 24 h). Cells were fixed with 4% paraformaldehyde for 15 min at room temperature, permeated with 0.1% Triton-X for 15 min before being blocked in 0.3% BSA for 20 min. Samples were then incubated for 3 h with primary rabbit anti-4-HNE (Cat#HNE11-S, Alpha Diagnostic Int., San Antonio, TX, USA) or mouse anti-Nrf2 (Abcam, Cambridge, UK) and rabbit anti-HO-1 (Stressgen, Ann Arbor, MI, USA) antibodies diluted 1:100 in 0.3% BSA in PBS. At the end of incubation, all samples were washed twice in PBS and incubated at room temperature for 90 min, light-protected, with secondary antibodies diluted 1:1000 in PBS. Goat anti-rabbit 488 (Alexa Fluor) was used for 4-HNE and HO-1, whereas donkey anti-mouse 546 (Alexa Fluor) was used to detect Nrf2 labeling. Moreover, cell nuclei were counterstained with DAPI (1:1000 in PBS) for 10 min at room temperature, in a light-protected environment. Subsequently, the samples were coverslipped with an antifade medium (ProLong Gold; Invitrogen). Images (20×) were obtained with a confocal laser scanning system (TCSSP2, Leica) equipped with an Ar/ArKr laser (for 488 nm excitation) and HeNe laser (for 543 nm excitation). DAPI staining was imaged by two photon excitations (740 nm, <140 fs, 90 MHz) performed with an ultrafast, tunable mode-locked Ti:sapphire laser (Chameleon; Coherent Inc., Santa Clara, CA, USA).

Concerning Nrf2 and HO-1 immunofluorescence, 20 μm-thick confocal *Z*-stacks in series were acquired as images of 1024 pixels × 1024 pixels, reordered at intervals of 0.5 μm, in order to evaluate the real extent of the nuclear and/or cytosolic fluorescence in high magnifications (63×).

The optical density analysis to quantify fluorescence signal was performed by using Leica Confocal Software (LCS Lite); each evaluation was conducted on five fields randomly selected for each of the experimental conditions.

### DNA Fragmentation

DAPI staining was used to identify nuclear fragmentation and condensed nuclei in SH-SY5Y cells. Briefly, 300,000 cells/glass were fixed with 4% paraformaldehyde for 15 min, washed twice in PBS and then incubated in a DAPI containing solution (1:1000 in PBS 0.1 M) and Triton (TX, 0.1% in PBS 0.1 M) for 10 min at room temperature and light-protected. Samples were, then, washed in PBS and coverslipped with an antifade medium (ProLong Gold; Invitrogen). Images were taken at low (20×) and higher magnification (40×) by a confocal laser scanning microscope (TCS-SP2; Leica Microsystem, GmbH, Wezlar, Germany). To highlight changes in morphology like nuclear condensation and cellular fragmentation, DAPI staining was imaged by two-photon excitation (740 nm, <140 fs, 90 MHz) performed by an ultrafast tunable mode-locked titanium:sapphire laser.

### Superoxide Anion Production

The content of intracellular superoxide anion was determined by laser scanning confocal microscope analysis with superoxide-sensitive fluorescent probe dihydroethidium (DHE, Invitrogen Molecular Probes). Briefly, 300,000 cells/glass in different culture conditions (see Results) were incubated with DHE (10 μM) at 37°C for 30 min in the dark. The cells were washed twice with PBS and images were taken by a confocal laser scanning microscope (TCS-SP2; Leica Microsystem, GmbH, Wezlar, Germany) using two-photon excitation (792 nm, >140 fs, 90 MHz) performed by an ultrafast tunable mode-locked titanium:sapphire laser (Chameleon; Coherent). The optical density analysis was performed to quantify superoxide anion fluorescence signals by using LCSLite, Leica Confocal Software.

### Statistical Analyses

Results are presented as mean ± standard error of the mean (SEM) of *N* replicates per group. Data sets have been analyzed by One-way ANOVA corrected by the Dunnet or Newman–Keul tests for comparison within the same group or more groups, respectively (Prism 4.0 software, GraphPad Software). Differences were considered significant at *P* < 0.05.

## Results

### TMT Activates the HO-1/BVR System

**FIGURE 1:**
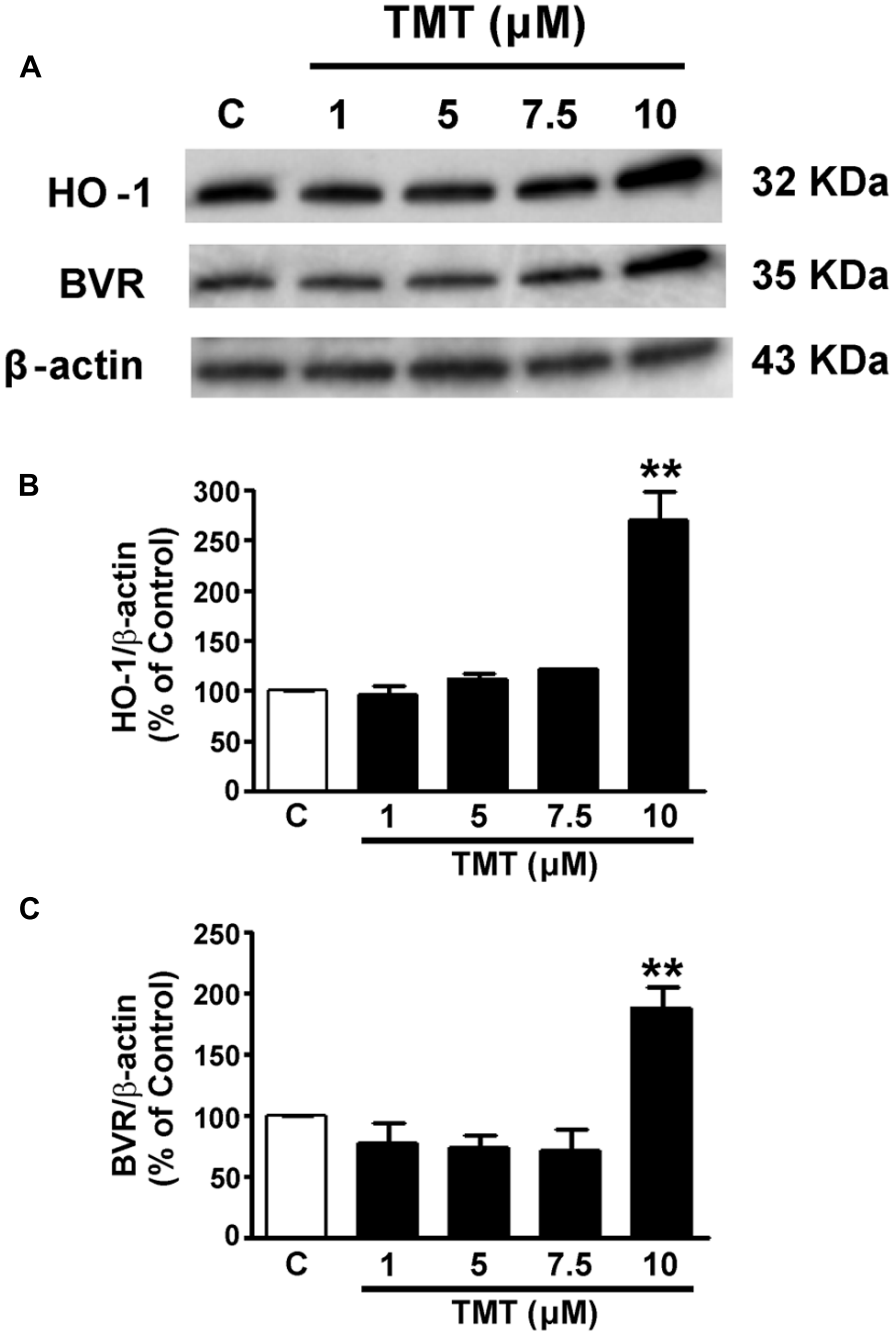
**Trimethyltin (TMT) increases both heme oxygenase-1 (HO-1) and biliverdin reductase (BVR) expression in SH-SY5Y cells.** SH-SY5Y cells were treated with TMT (1–10 μM) for 24 h and Western Blot was performed as described in the section “Materials and Methods.” **(A)** shows representative gels regarding HO-1 and BVR protein expression. Bar graphs represent the quantification of HO-1 **(B)** and BVR **(C)** protein levels normalized for β-actin expression. Data are expressed as a mean ± SEM of 4–7 replicates per group. ^∗∗^*P* < 0.01 vs. controls. C, controls.

Since only one work has been found in literature concerning the effect of TMT on HO-1, in a preliminary series of experiments, it was necessary to study the effect of TMT on the expression of HO-1 and BVR in SH-SY5Y cells. The incubation time of 24 h of the cells with TMT was chosen since literature data have shown that the production of ROS and the consequent oxidative stress continue during the 24 h following the treatment of primary cultures of rat cerebellar neurons with this neurotoxicant (Gunasekar et al., 2001). As shown in **Figure 1**, TMT (1–10 μM for 24 h) dose-dependently increased HO-1 and BVR expression. However, only at the concentration of 10 μM, TMT increased both HO-1 and BVR protein expression reaching statistical significance. In light of this, TMT was used at the concentration of 10 μM for 24 h in the subsequent experiments.

### FA-Induced Modulation of HO-1/BVR in TMT-Treated SY5Y Cells

**FIGURE 2:**
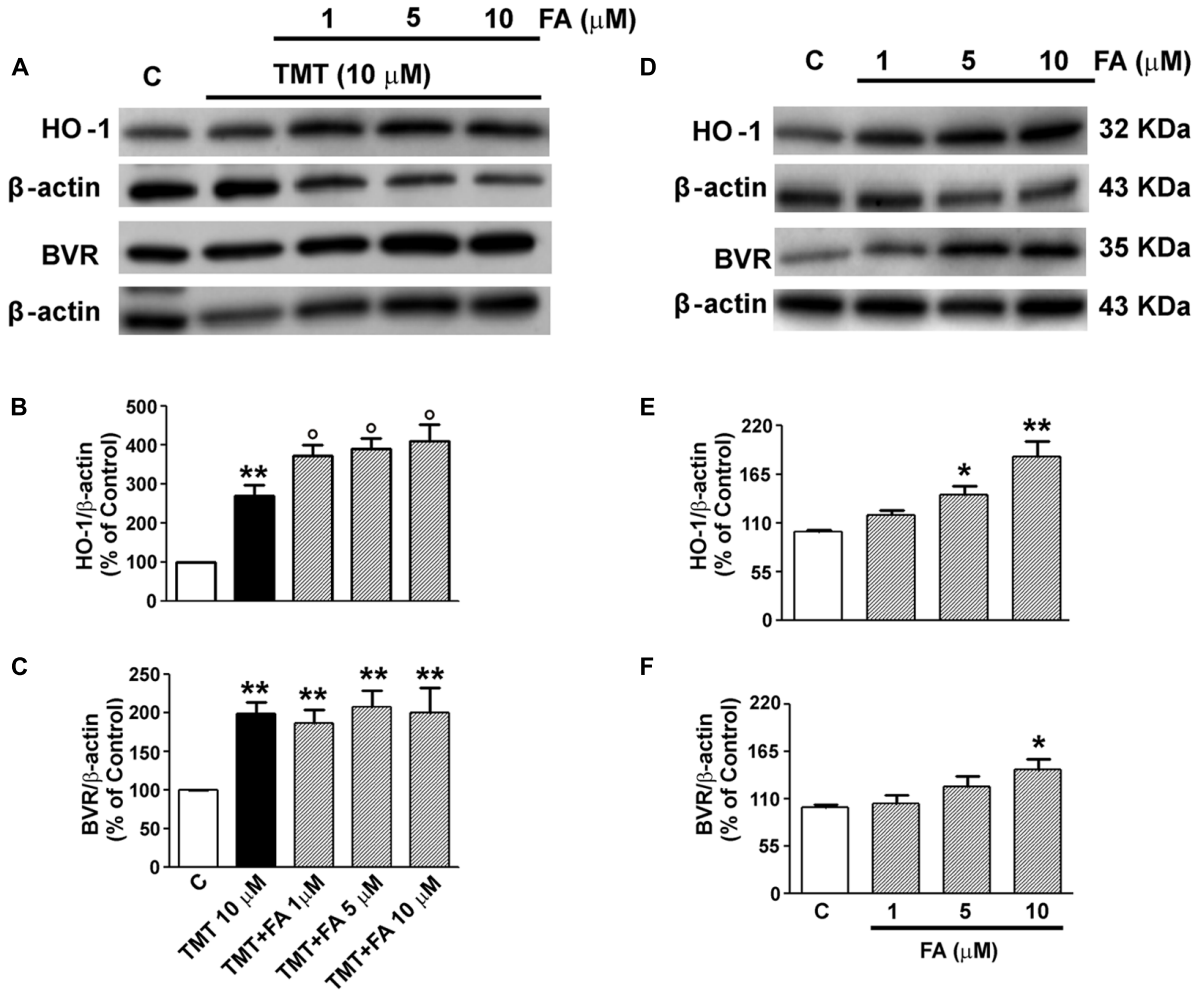
**Ferulic acid (FA) effects on TMT-induced heme oxygenase-1 (HO-1) and BVR expression in SH-SY5Y cells.** SH-SY5Y cells were treated with FA (1–10 μM) for 6 h and then incubated with TMT (10 μM) for further 24 h **(A–C)**; in another group of experiments SH-SY5Y cells were treated with FA (1–10 μM) for 6 h and, subsequently, incubated with plain medium for additional 24 h **(D–F)**. Following incubation, cells were harvested and Western Blot was performed as described in the section “Materials and Methods.” **(A)** and **(D)** show representative gels regarding HO-1 and BVR protein expression. Bar graphs represent the quantification of HO-1 **(B,E)** and BVR **(C,F)** protein levels normalized for β-actin expression. Data are expressed as a mean ± SEM of eight replicates per group. ^∗^*P* < 0.05 and ^∗∗^*P* < 0.01 vs. controls, °*P* < 0.05 vs. TMT. C, controls.

As regards FA, Sultana et al. (2005) have shown its ability to increase the expression of HO-1 as early as 1 h of incubation in cortical neurons. However, time-course experiments carried out specifically for this study, have shown it takes at least 6 h of incubation of SH-SY5Y cells with FA for a significant induction of HO-1 (data not shown) and, for this reason, in subsequent experiments, cells were incubated with FA for 6 h, also in accordance with previous studies (Scapagnini et al., 2004). The range of FA doses, namely 1–10 μM, was decided taking into consideration the magnitude of plasma concentration of FA in humans and also according to previous studies that have allowed the calculation of the EC_50_ of this antioxidant in reducing the ROS/RNS damage in rat brain microsomes (Barone et al., 2009a; Trombino et al., 2013; Mancuso and Santangelo, 2014). As shown in **Figures 2A–C**, FA (1–10 μM) significantly enhanced TMT-induced HO-1 expression in SH-SY5Y cells, whereas the antioxidant did not have any relevant effect on TMT-induced BVR expression. Interestingly, FA (1–10 μM) also increased, in a dose-dependent manner, basal HO-1 and BVR expression in SY5Y cells (**Figures 2D–F**).

**FIGURE 3:**
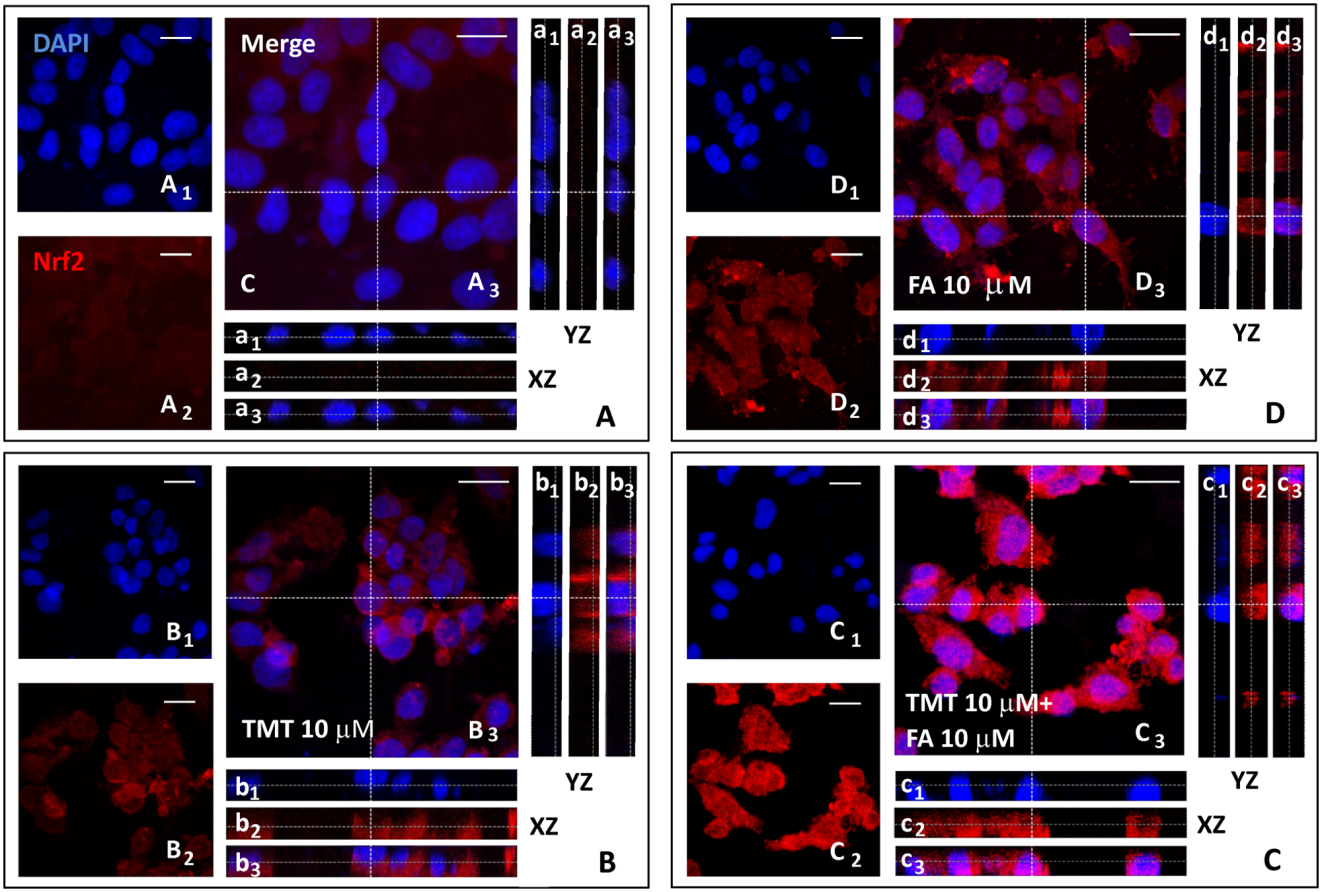
**Ferulic acid induces Nrf2 activation and translocation into the nucleus. (A–D)** Representative images from four independent immunofluorescence experiments in which we performed a double-labeling with DAPI **(A_1_–D_1_)** and an anti-Nrf2 antibody **(A_2_–D_2_)**. Merged images are shown in **(A_3_–D_3_)**. XZ and YZ cross-sections in the boxes (referred to the dashed lines) from the confocal *Z*-stack acquisitions illustrate cytosolic or nuclear fluorescence signal(s) (XZ and YZ boxes: a_1_–d_1_ refer to DAPI staining; a_2_–d_2_: refer to Nrf2 fluorescence; a_3_–d_3_: Merge). In cells treated with TMT, a strong Nrf2 activation was detected which, however, remains mainly confined in the cytoplasm (b_1_–b_3_ in **B)**. In TMT + FA treated cells, there was a further cytoplasmic enhancement of Nrf2 expression compared to TMT alone which also translocates into the nucleus as indicated by *Z*-stack acquisitions (c_1_–c_3_ in **C)**. FA administration (10 μM) induced an endogenous antioxidant response leading to a strong increase in Nrf2 expression in the cytosol (d_1_–d_3_ in **D)** compared to Control (a_1_-a_3_ in **A)**. Scale bar: 20 μm. For further information see text.

**FIGURE 4:**
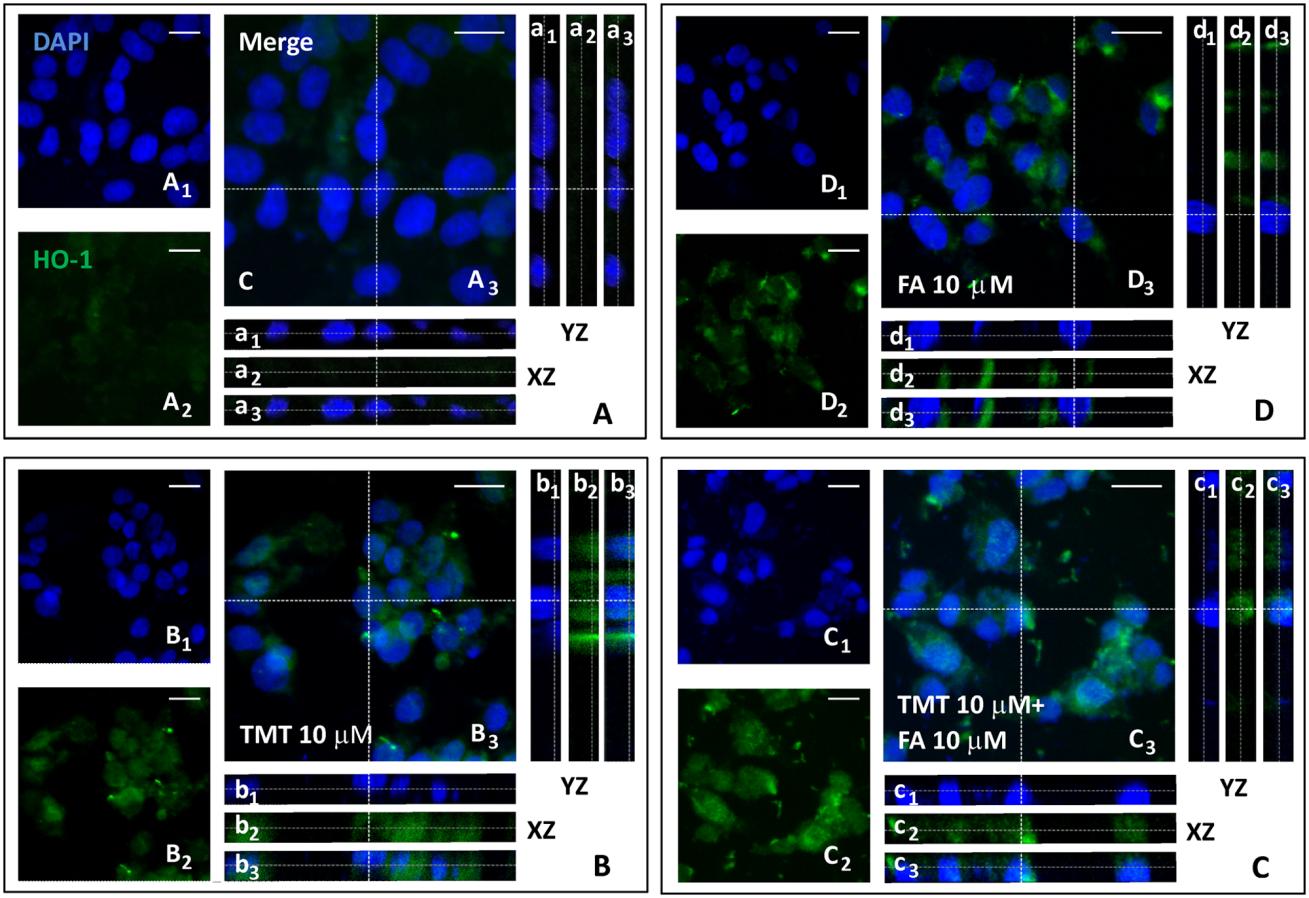
**Ferulic acid induces HO-1 expression and its nuclear translocation. (A–D)** Representative images from four independent immunofluorescence experiments in which we carried out a double-labeling with DAPI **(A_1_–D_1_)** and an anti-HO-1 antibody **(A_2_–D_2_)**. Merged images are shown in **A_3_–D_3_**. XZ and YZ cross-sections in the boxes (referred to the dashed lines) from the confocal *Z*-stack acquisitions show cytosolic or nuclear fluorescence signal(s) (XZ and YZ boxes: a_1_–d_1_ refer to DAPI staining; a_2_–d_2_ refer to HO-1 fluorescence; a_3_–d_3_: Merge). In the Control group **(A)** a faint HO-1 fluorescence localized in the cytosol was detected (a_1_–a_3_). In TMT (10 μM) treated cells **(B)** HO-1 fluorescence was strong and primarily localized in the cytoplasm (b_1_–b_3_). In TMT (10 μM) + FA (10 μM) samples **(C)** a marked HO-1 activation both in cytoplasm and nucleus was observed, as indicated by *Z*-stack acquisitions (c_1_–c_3_). FA (10 μM) treatment induced a strong or moderate increase of HO-1 labeling, localized in the cytoplasm and nucleus, respectively (d_1_–d_3_ in **D)**. Scale bar: 20 μm. For further information see text.

Since one of the main mechanisms by which HO-1 is expressed is the translocation of the transcription factor Nrf2, from the cytoplasm to the nucleus, where it binds the antioxidant responsive element (ARE) region of the gene promoter HO-1, one of the goals of the study was to investigate whether the increased expression of HO-1, induced by FA, had depended on the nuclear translocation of Nrf2 in SH-SY5Y cells. **Figures 3** and **4** shows representative Nrf2 (red fluorescence) and HO-1 (green fluorescence) immunostainings of SY5Y cells treated with TMT (10 μM) and FA (10 μM), i.e., the most effective concentrations that modulate HO-1. The intensity of fluorescence signal for Nrf2 and HO-1 was quantified by measuring the optical density in the cytosol and nucleus of SH-SY5Y and, afterwards, the cytosol/nucleus ratio (C/N) was calculated. In control samples, Nrf2 was mainly displaced in the cytosol (**Figure 3A**) and a faint HO-1 expression was observed in this compartment (**Figure 4A**, **Table 1**) as shown by the XZ and YZ cross-sections from the *Z*-stack acquisitions (boxes a_1_–a_3_ in **Figures 3A** and **4A**, respectively). A moderate-to-strong increase in Nrf2 fluorescence (**Figure 3B**, **Table 1**) was detected in TMT-treated cells, mainly located inside the cytosol, although *Z*-stack and optical density analysis showed a comparable nuclear accumulation of the transcription factor (boxes b_1_–b_3_) in SY5Y cells. Simultaneously, a similar enhancement in HO-1 (**Figure 4B**, **Table 1**) expression confined inside the cytosol (boxes b_1_–b_3_) was observed in the same samples. The combined treatment with TMT and FA further increased Nrf2 expression (**Figure 3C**, **Table 1**) with respect to TMT alone. As clearly indicated by XZ and YZ cross-sections, the Nrf2 fluorescence signal was further increased, not only in the cytosol, but also in the nucleus, in SY5Y cells exposed to TMT + FA (boxes c_1_–c_3_ in **Figure 3C**, **Table 1**). Interestingly, SY5Y cells treated with TMT + FA showed also a marked over-expression of HO-1 (**Figure 4C**, **Table 1**), both in the cytosol and in the nucleus (boxes c_1_–c_3,_ XZ and YZ cross-sections). The administration of FA (10 μM) induced a strong activation of Nrf2, which translocated into the nucleus, as shown by the XZ and YZ cross-sections (boxes d_1_–d_3_ **Figure 3D**, **Table 1**). Moreover, a remarkable increase of HO-1 labeling was observed, primarily inside the cytosol whereas a moderate immunostaining was also detected in the nucleus (boxes d_1_–d_3_ **Figure 4D**, **Table 1**). The data in **Table 1** allow to conclude that the enhancement of Nrf2 expression and its translocation into the nucleus parallels the up-regulation of HO-1 protein expression under all the experimental conditions; however, FA and TMT mainly increased the cytosolic HO-1 (C/N 3.67 and 2.69, respectively) whereas FA + TMT increased HO-1 immunostaining in the cytosol but much larger into the nucleus (C/N 1.10).

**Table 1:** Nrf2 and HO-1 fluorescence signals (average optical density values from four independent experiments) in the cytosol and nucleus of SH-SY5Y cells.

	Nrf2			HO-1		
	Cytosol	Nucleus	C/N	Cytosol	Nucleus	C/N
Control	18.5	9.33	1.98	11.1	6.67	1.66
TMT 10 μM	72.4	42.4	1.70	36.4	13.5	2.69
TMT 10 μM + FA 10 μM	97.7	83.3	1.17	95.7	87.0	1.10
FA 10 μM	69.0	74.7	0.92	62.4	17	3.67

*C/N, cytosol/nucleus ratio.*

### FA-Regulated Lipid Peroxidation and DNA Fragmentation in TMT-Treated SY5Y Cells

**FIGURE 5:**
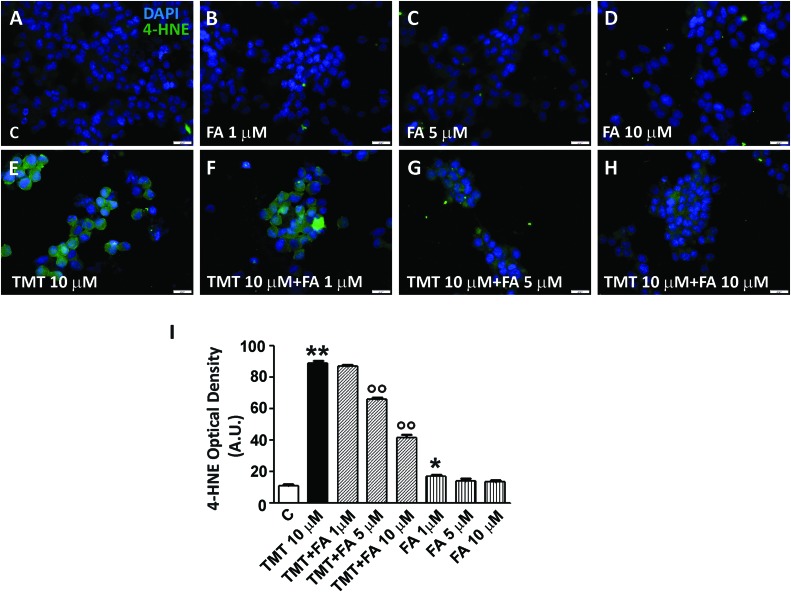
**Ferulic acid counteracts the increase of lipid peroxidation induced by TMT in a dose-dependent manner. (A–H)** Representative immunofluorescence images of SH-SY5Y cells treated with TMT (10 μM) and/or FA (1–10 μM) and double stained with DAPI (blue) and anti-4-HNE antibody (green). 4-HNE labeling was faint in control cells **(A)**, but mildly increased in cells treated with different doses of FA **(B–D)**. After TMT treatment, there was a marked increase of 4-HNE fluorescence **(E)** that decreased in a dose-dependent manner when FA was added **(F–H)**. **(I)** Histograms (mean ± SEM) showing optical density quantification in treated cells for 4-HNE labeling. Data are representative of three independent experiments; each count was performed on five fields randomly selected for each experimental condition. Scale bar **(A–H)** 20 μm. ^∗^*P* < 0.05, ^∗∗^*P* < 0.01 vs. controls **(C)**, ^∘∘^*P* < 0.01 vs. TMT.

**FIGURE 6:**
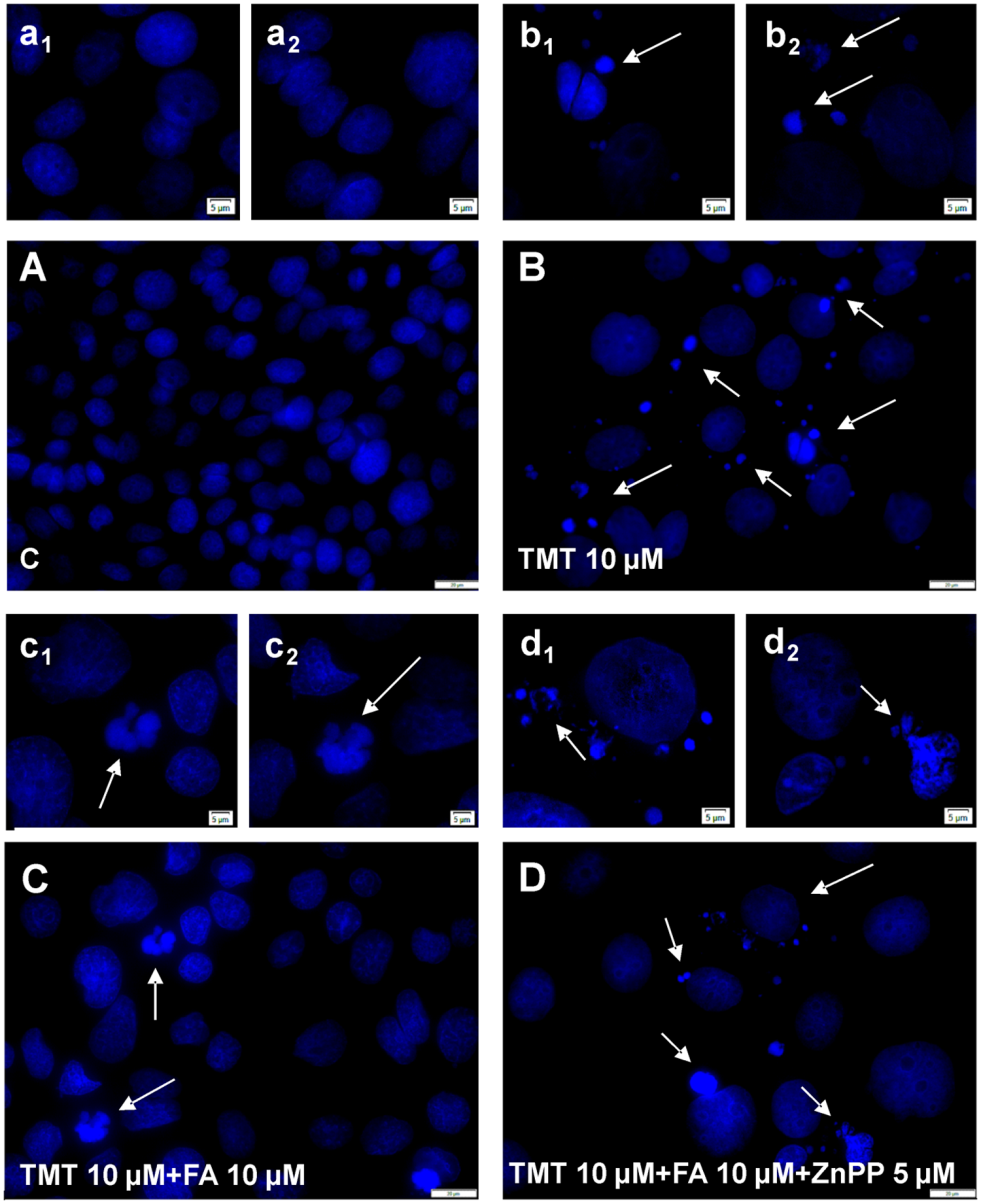
**Ferulic acid neutralizes TMT-induced DNA fragmentation through the modulation of HO activity.** Representative immunofluorescence images of SH-SY5Y cells stained with DAPI at low magnification (20×, **A–D)** and high magnification (40×, a_1_–d_2_). Control cells **(A)** showed no morphological signs of DNA fragmentation. TMT (10 μM, **B)** induced morphological signs of cytotoxicity such as nuclei condensation and nuclear fragmentation (see arrows). This effect was counteracted by FA (10 μM, **C)** pre-treatment. When HO activity was inhibited by ZnPP (5 μM) in TMT (10 μM) + FA (10 μM) treated cells **(D)**, nuclear fragmentation and condensed nuclei were detected, with no difference compared to TMT treatment (see arrows). Scale bar: **(A–D)** 20 μm; a_1_–d_2_: 5 μm. For further information see text.

Among the most common ways by which free radicals irreversibly damage cells are lipid peroxidation and DNA damage. 4-hydroxy-nonenals (4-HNE) are widely used as biomarkers of lipid peroxidation also because they are considered very cytotoxic products (Subramaniam et al., 1997). As far as DNA damage, free radicals are responsible for single- or double-strand breaks, tandem lesions and other types of adducts which, ultimately, lead to DNA fragmentation (Dizdaroglu and Jaruga, 2012). As shown in **Figure 5**, TMT (10 μM for 24 h) markedly increased 4-HNE levels in SH-SY5Y cells; when the latter were pre-treated with FA (1–10 μM for 6 h), TMT-induced 4-HNE formation was reduced in a dose dependent manner, reaching significant values at a concentration of FA 5 μM and further decreasing to 10 μM. Interestingly, FA (1–10 μM) mildly increased basal 4-HNE formation, reaching statistical significance at 1 μM. With regard to DNA damage, TMT (10 μM for 24 h) clearly increased DNA fragmentation in SH-SY5Y cells (**Figure 6B**) and this effect was counteracted by FA (10 μM for 6 h; **Figure 6C**).

**FIGURE 7:**
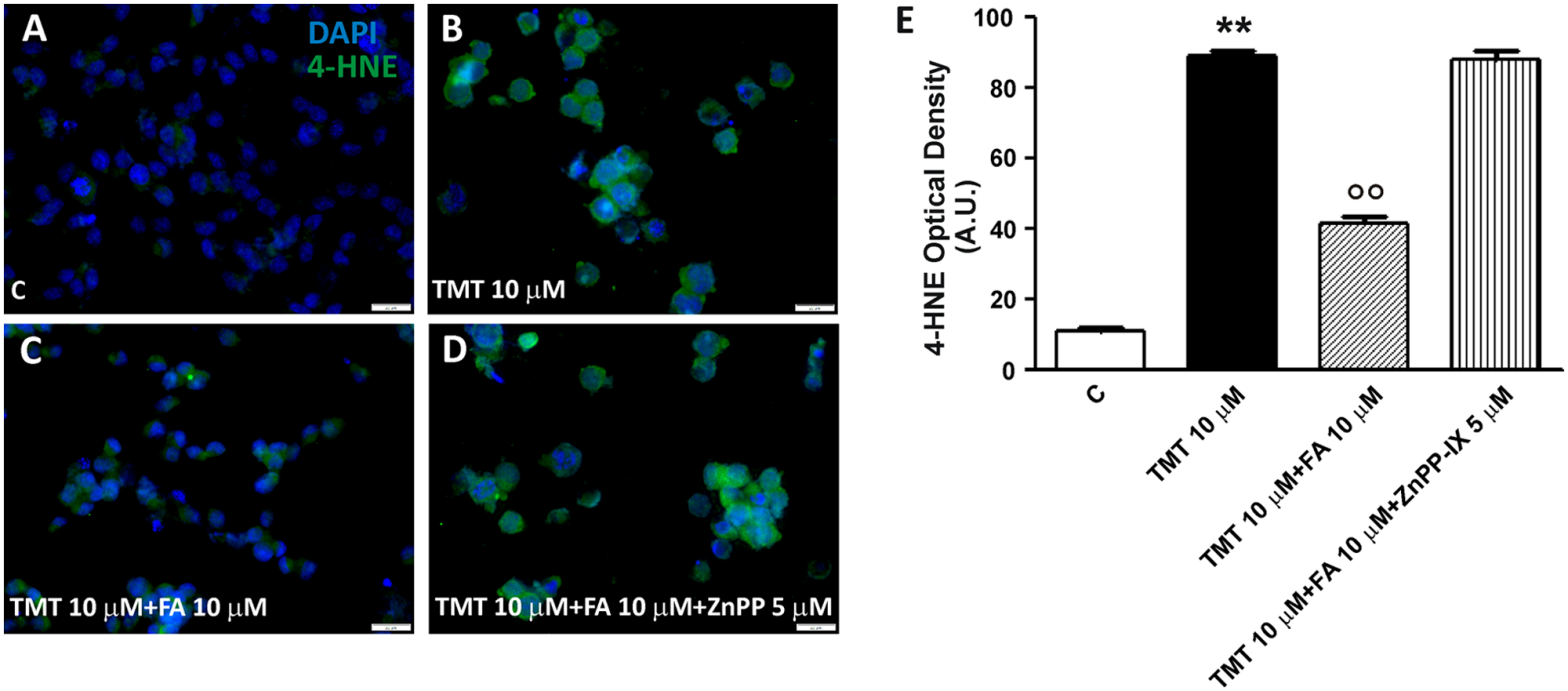
**Ferulic acid inhibits lipid peroxidation through the HO activity. (A–D)** Representative immunofluorescence images of SH-SY5Y cells treated with TMT (10 μM), FA (10 μM), and ZnPP-IX (5 μM) double stained with DAPI (blue) and an anti-4-HNE antibody (green). 4-HNE labeling was faint in control cells **(A)**. After TMT treatment, a remarkable increase of 4-HNE fluorescence was detected **(B)**. The combined treatment with TMT and FA decreases significantly lipid peroxidation **(C)** whereas, when cells were treated with TMT and FA and ZnPP, 4-HNE labeling remains marked similarly to the result observed in TMT treated cells **(D)**. **(E)** Bar graphs (mean ± SEM) showing optical density quantification in treated cells for 4-HNE labeling. Data are representative of three independent experiments; each count was performed on five fields randomly selected for each experimental condition. Scale bar **(A–D)** 20 μm. For further information see text. ^∗∗^*P* < 0.01 vs. controls **(C)**, ^∘∘^*P* < 0.01 vs. TMT.

SH-SY5Y cells were treated with ZnPP – a well known inhibitor of HO activity – in order to confirm that FA counteracted TMT-induced 4-HNE formation and DNA fragmentation by modulating HO activity. At the concentration of 5 μM, ZnPP has antagonized both the reduction of the 4-HNE levels (**Figure 7**) and DNA fragmentation (**Figure 6D**) caused by 10 μM FA, thus confirming the hypothesis that the antioxidant action of FA is connected to the HO activity. It is worth emphasizing that the concentration of ZnPP, used is well below the concentration limit at which it remains selective concerning HO without any effect on other heme proteins, such as NOS and sGC (Appleton et al., 1999).

**FIGURE 8:**
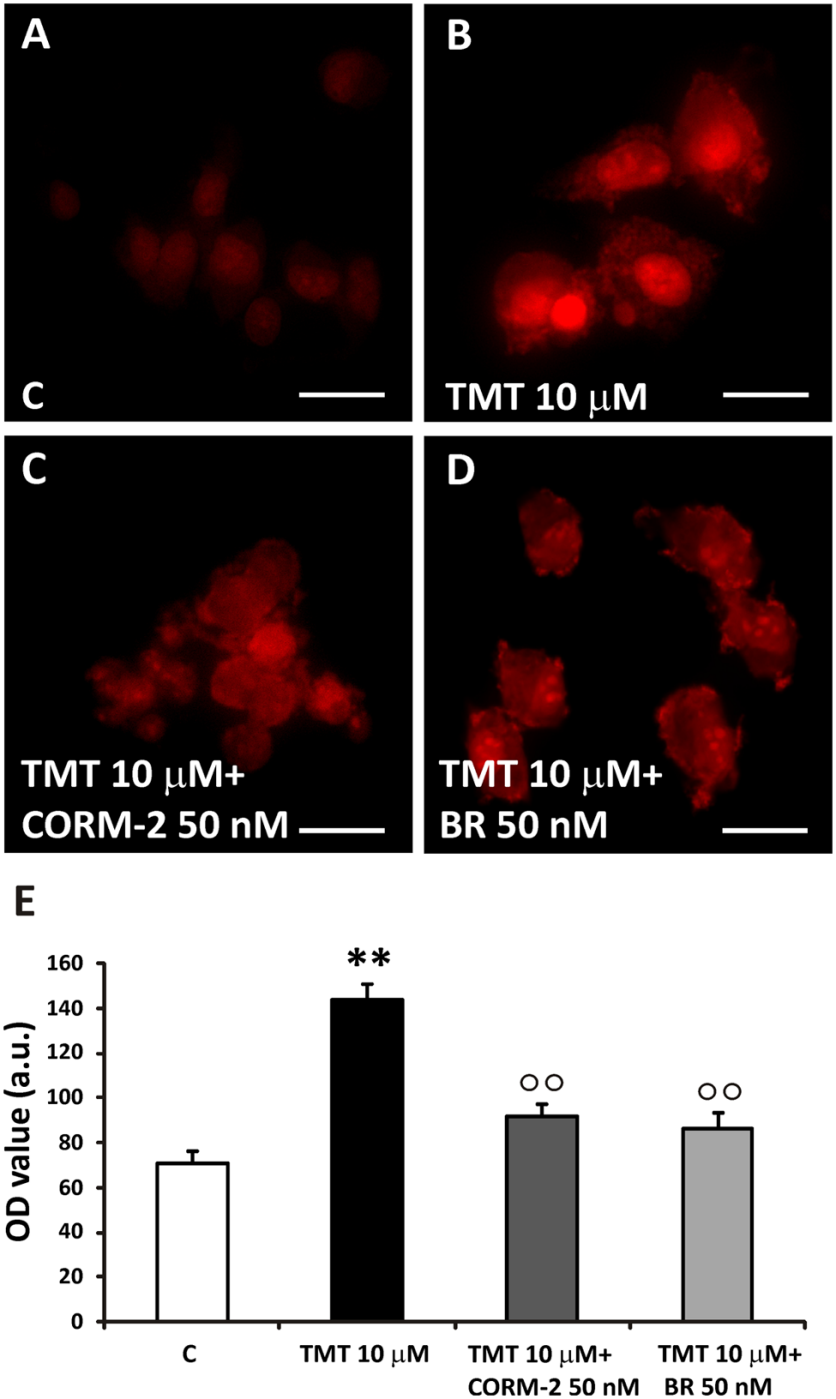
**TMT-induced superoxide production. (A–D)** Representative immunofluorescence images of SH-SY5Y cells treated with TMT (10 μM), CORM-2 (50 nM) and bilirubin (BR, 50 nM) and stained with DHE. In control cells, DHE staining was faint **(A)**. TMT (10 μM) induces a great increase in superoxide production, as indicated by the rise of red fluorescence **(B)**. When cells are treated with TMT (10 μM) + CORM-2 (50 nM; **C)** or TMT (10 μm) + BR (50 nM; **D)**, ROS production significantly decreases with respect to TMT alone. In **(E)**, a bar graph (mean ± SEM) showing fluorescence signal quantification in treated cells for 4-HNE labeling. Data are representative of three independent experiments performed on five field selected randomly. Scale bar: 20 μm. Magnification: 40×. For further information see text. ^∗∗^*P* < 0.01 vs. controls **(C)**, ^∘∘^*P* < 0.01 vs. TMT.

With the aim to evaluate which by-product of the HO/BVR activities was involved in the FA’s antioxidant features, the effects of both CORM-2 (a CO donor) and BR against TMT-induced superoxide anion production were tested in SH-SY5Y cells. The major concern we had to face with, while designing these experiments, was the choice of both BR and CORM-2 concentrations so that they could not result toxic when co-administered with TMT. In order to overcome this issue, we decided to use BR (50 nM), a concentration found safe for SH-SY5Y cells by Dal-Cim et al. (2012); CORM-2 was used at the same concentration of BR for proper comparison. In this experimental setting SH-SY5Y cells were incubated with either BR (50 nM) or CORM-2 (50 nM) for 6 h and then the culture medium replaced with fresh medium containing TMT (10 μM) plus BR or CORM-2, as above, for further 24 h. In an attempt to maintain a continuous release of CO, CORM-2-containing cell culture media were replaced every 6 h. As shown in **Figure 8**, CORM-2 (50 nM) and BR (50 nM) significantly reduced TMT (10 μM)-induced superoxide anion generation in SH-SY5Y cells. Neither BR nor CORM-2 had any significant effect on basal superoxide anion production (data not shown).

## Discussion

Very little evidence has proven the effects of FA in the event of brain damage due to the action of neurotropic xenobiotics, such as the neurotoxin TMT, despite the large number of studies showing the protective efficacy of FA in synaptosomes or neural cells exposed to free radical generators, including Aβ (Kanski et al., 2002; Joshi et al., 2006; Perluigi et al., 2006). Only one study is present in the literature on this topic and it identifies the modulation of choline acetyl-transferase as effector of the long-term neuroprotective activity of FA on TMT-induced cognitive impairment in mice (Kim et al., 2007). However, in their paper, Kim et al. (2007) did not evaluate the role of FA on TMT-induced free radical generation and redox imbalance which are considered important mechanisms in the pathogenesis of TMT damage in neural cells (Corvino et al., 2013). Bearing this in mind, we decided to open this new route and study the effect of FA on TMT-induced oxidative damage in SH-SY5Y, a human neuronal cell line widely used for research on the mechanisms of neurodegeneration/neuroprotection (Li et al., 1996; Bhat et al., 2000; Uberti et al., 2002). Furthermore, we also investigated the neuroprotective effectiveness of the HO-1/BVR system -which plays a prominent role in the cellular stress response- on TMT neurotoxicity.

One of the first results to be discussed is that, when the SH-SY5Y cells were treated with FA (1–10 μM) in the presence of TMT (10 μM), the former has significantly and dose-dependently enhanced the activation of HO-1 by the latter (**Figures 2A,B**). This finding shows that, under pro-oxidants conditions, such as those generated by the administration of TMT (**Figures 5** and **6**), FA further increases the expression of HO-1, yielding a particularly marked result at the concentration of 10 μM FA. FA had no effect on the expression of BVR induced by TMT (**Figures 2A,C**). Interestingly, FA (1–10 μM) increased, in a dose-dependent manner, the expression of HO-1 and BVR also in SH-SY5Y cells not treated with TMT (**Figures 2D–F**), confirming its nature of hormetin (Barone et al., 2009a). Indeed, 1 μM FA had a type of pro-oxidant effect on SH-SY5Y cells, as demonstrated by the significant increase of 4-HNE, and this event may have triggered the initial overexpression of HO-1 (+18% vs controls; **Figures 2D,E** and **5**). At doses of 5 μM and 10 μM FA, the induction of HO-1 in SH-SY5Y cells further increased (+42 and +84% vs. controls, respectively) and the production of 4-HNE progressively decreased (**Figures 2D,E** and **5**). Therefore, the increased basal expression of HO-1, may have start a particular antioxidant “prophylactic” action by FA in a subsequent attack due to an excessive production of free radicals.

The mechanism by which FA induced HO-1, both in basal conditions and in the presence of TMT, is linked to the activation of Nrf2 and its translocation from cytosol to the nucleus (**Figure 3**). This evidence is somewhat new in neural cells, as the effects of FA on the Nrf2/HO-1 axis have been studied only in lymphocytes and endothelial cells (Ma et al., 2010, 2011). A qualifying part of our study is that it also highlights the translocation of HO-1 from the cytosol to the nucleus in SH-SY5Y cells exposed to FA alone or in combination with TMT (**Figures 3** and **4**). Previous studies have shown the nuclear translocation of HO-1 in human prostate cancer cells, murine fibroblasts and mouse hepatoma cells exposed to strong oxidant conditions, such as cigarette smoke or hypoxia or hemin with or without hemopexin (Lin et al., 2007; Sacca et al., 2007; Birrane et al., 2013). In all these studies, the presence of HO-1 in the nucleus, due to strong pro-oxidant stimuli, was considered a functional mechanism which could enhance the transcription of other cytoprotective genes able to counteract oxidative damage. In the present study, FA-induced HO-1 overexpression seems to be related mainly to the Nrf2 translocation from the cytosol to the nucleus, whereas when the antioxidant was co-administered with TMT not only increases Nrf2-stimulated cytosolic HO-1, but this condition favors HO-1 nuclear translocation, arguably with the purpose to enhance the antioxidant response. The question if and which other neuroprotective genes may have been induced by FA through the nuclear translocation of HO-1 and their possible effect is outside the scope of this work, which, however, has fully addressed the antioxidant effectiveness of FA through the enzymatic activity of HO. Indeed, FA (1–10 μM) significantly reduced, in a dose-dependent fashion, TMT-induced 4-HNE generation and DNA fragmentation in SH-SY5Y cells and this effect was counteracted by the HO inhibitor ZnPP (**Figures 5–7**). Evidence -suggesting that the ZnPP has abolished the antioxidant effects of FA bringing the production of 4-HNE and DNA damage to levels corresponding to those caused by the administration of TMT- indicates that, in our experimental system, the antioxidant activity of FA is almost completely due to the activation of HO-1 and its effect on free radicals. The result about a significant antioxidant activity for FA against TMT-induced neurotoxicity, together with the finding of a marked nuclear translocation of HO-1 under this experimental condition (C/N 1.10), reinforce each other and lend support to the hypothesis that HO-1 nuclear translocation is critical for the neuroprotective activity of FA.

With regard to the “ultimate protectant” responsible for the FA’s antioxidant effect, both CORM-2 and BR, at low concentrations, significantly reduced TMT-induced superoxide anion production in SH-SY5Y cells (**Figure 8**). That said, it is noteworthy to recall also the importance of heme reduction as an additional antioxidant mechanism.

## Conclusion

Our studies have shown that FA plays neuroprotective effects on cell lines of human neurons through the activation of the HO-1/Nrf2 system, probably via CO and BR production, and how it is responsible for reducing the oxidizing effects of the neurotoxin, TMT.

## Author Contributions

SC, FP, FM, and RR carried out the experiments. DT and VC contributed to analyze the data and provided a critical reading of the manuscript. RS and CM designed the experiments, analyzed the data, interpreted the results and wrote the paper. CM provided funds.

## Conflict of Interest Statement

The authors declare that the research was conducted in the absence of any commercial or financial relationships that could be construed as a potential conflict of interest.
